# The complete mitochondrial genome of *ostorhinchus fleurieu* (kurtiformes: Apogonidae) and phylogenetic studies of apogoninae

**DOI:** 10.1080/23802359.2019.1679681

**Published:** 2019-10-23

**Authors:** Kehua Zhu, Yuanpei Gao, Pengxiang Yuan, Pinglin Cao, Xiaoguo Ying, Hengcong Tao, Bingjian Liu

**Affiliations:** a National engineering Laboratory of Marine Germplasm Resources Exploration and Utilization, Zhejiang Ocean University, Zhoushan, China;; b Key Laboratory of Informatization of Habitat Monitoring and Fishery Resource Conservation Research in the East China Sea of Zhejiang Province, Zhejiang Ocean University, Zhoushan, China;; c Key Laboratory of Aquatic Products Processing of Zhejiang Province, Department of Food Science and Pharmacy, Zhejiang Ocean University, Zhoushan, China;; d Laboratory for Marine Fisheries Science and Food Production Processes, Qingdao National Laboratory for Marine Science and Technology, Qingdao, China

**Keywords:** *Ostorhinchus fleurieu*, mitogenome, phylogenetic position

## Abstract

The complete mitochondrial genome of *Ostorhinchus fleurieu* was first determined, which was 16,521 bp in length, containing 13 protein-coding genes, two rRNA genes, 22 tRNA genes, a putative control region and one origin of replication on the light-strand. The overall base composition included C(29.2%), A(26.7%), T(26.7%) and G(17.4%). Moreover, the 13 PCGs encoded 3800 amino acids in total, twelve of which used the initiation codon ATG except for COI started with GTG. Most of them ended with complete stop codon, whereas three protein-coding genes (COII, ND4 and Cytb) used incomplete stop codon and represented as T. The phylogenetic tree based on the Neighbour Joining method was constructed to provide relationship within Apogoninae, which could be a useful basis for management of this species.

The flower cardinalfish (*Ostorhinchus fleurieu*) distributes in the Visakhapatnam waters, Middle East coast of India (Randall and Hayashi 1990), and it is common in shallow coastal reefs with moderate currents, also in tidal channels of estuaries (Fraser 2014). The genus *Ostorhinchus* is composed of nearly 60 species, however, the genetic and molecular information of *Ostorhinchus* is limited, plus there are no any complete mitogenomes of *Ostorhinchus* have been determined (Fraser 2014), these factors jointly contributed to the motivation of sequencing this species. To gain its molecular information, herein, we described the complete mitogenome of *O. fleurieu* and explored the phylogenetic relationship within Apogoninae, contributing to further phylogenetic studies on its related species.

Specimen was collected from Nandu river, Haina province, China (18°29′48″N; 109°56′12″E) and stored in the Molecular Breeding Laboratory of Zhejiang Ocean University with accession number 20150826SP22.

The mitogenome of *O*. *fleurieu* was a closed double-stranded circular molecule of 16,521bp (GenBank accession No. MN381712), which contained 13 PCGs, 2 rRNA and 22 tRNA genes, and 2 main non-coding regions; nucleotide composition of the complete mitogenome was biased toward A and T, with a slightly higher content of A + T (53.4%) than G + C; genes were not coded totally by a single, most of them located on the heavy strand while ND6 and eight tRNA genes (Gln, Ala, Asn, Cys, Tyr, Ser, Glu, Pro) were detected on the light strand, these features are identical to those of other vertebrates (Cui et al. 2009; Jing et al. 2010; Zhu et al. 2018a). 13 PCGs encoded 3,800 amino acids in total, all PCGs began with a canonical start codon NTG. For the stop codon, most of PCGs were terminated with the complete codon TAA, TAG or AGA, whereas three PCGs (COII, ND4 and Cytb) located immediately upstream of tRNAs ended with only T, some authors suggested that these truncated stop codons were caused by post-transcriptional polyadenylation (Ojala et al. 1981).

The small and large subunit ribosomal RNAs (12S and 16S) were 957 and 1687 bp, respectively. They were located in the typical positions between tRNA-Phe and tRNA-Leu^(UUA)^, separated by tRNA-Val. The origin of light-strand replication was located in a cluster of five tRNA genes (WANCY) as in other vertebrates (Zhu et al. 2018b, 2018c, 2018d), which has the capacity of folding into a stable stem-loop secondary structure; the CR was determined to be 854 bp, by comparing the sequences of the CR with other teleost, three typical domains were noticed, including termination-associated sequences, the central conserved sequence block domain and the conserved sequence block domain, which is identical to that in other teleostean mitogenomes (Ruokonen and Kvist 2002).

The results of phylogenetic analysis shown that *O. fleurieu* was closely related to the species of *Apogon*, with a highly supported value of 100% (Figure 1). The sequence and full annotation of *O. fleurieu* would provide more molecular resources about Apogon mitogenomes and lay a foundation for the further research involved with phylogenetic relationship within Apogoninae.

**Figure 1.**
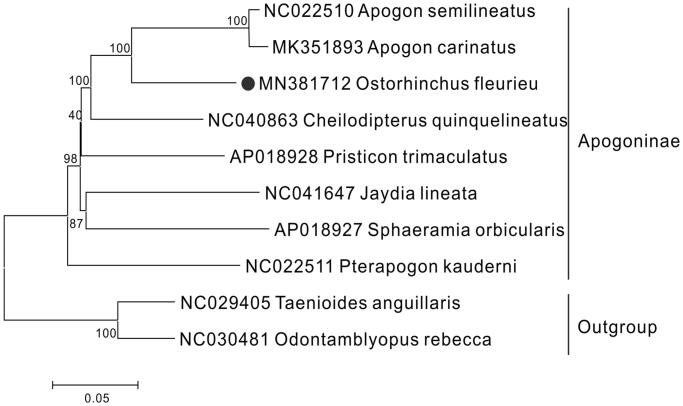
The phylogenetic tree of Ostorhinchus fleurieu and other 7 Apogoninae species was constructed using the Neighbour Joining (NJ) methods based on 12 protein-coding genes encoded by the heavy strand. The bootstrap values are based on 1,000 resamplings and the number at each node is the bootstrap probability. The number before the species name is the GenBank accession number. The genome sequence in this study is labelled with a black spot.
